# Silk-Corn Zein Alloy Materials: Influence of Silk Types (Mori, Thai, Muga, Tussah, and Eri) on the Structure, Properties, and Functionality of Insect–Plant Protein Blends

**DOI:** 10.3390/ijms26010186

**Published:** 2024-12-29

**Authors:** Nagireddy Poluri, Christopher R. Gough, Steven Sanderlin, Christopher Velardo, Anthony Barca, Joseph Pinto, Joseph Perrotta, Maxwell Cohen, Xiao Hu

**Affiliations:** 1 Department of Physics and Astronomy, Rowan University, Glassboro, NJ 08028, USA; poluri34@students.rowan.edu (N.P.);; 2 Department of Chemistry and Biochemistry, Rowan University, Glassboro, NJ 08028, USA; 3 Department of Biological and Biomedical Sciences, Rowan University, Glassboro, NJ 08028, USA

**Keywords:** silk fibroin, corn zein, composite film, secondary structure, biopolymer, protein–protein interaction

## Abstract

Biocompatible materials fabricated from natural protein polymers are an attractive alternative to conventional petroleum-based plastics. They offer a green, sustainable fabrication method while also opening new applications in biomedical sciences. Available from several sources in the wild and on domestic farms, silk is a widely used biopolymer and one of the strongest natural materials. This study aims to compare five different types of silk (Mori, Thai, Muga, Tussah, and Eri) fabricated into thin composite films in conjunction with plant-based proteins. To offer a wider range of morphologies, corn zein, another widely available protein material, was introduced into the silk protein networks to form blended polymers with various ratios of silk to zein. This resulted in the successful alloying of protein from an animal source with protein from a plant source. The material properties were confirmed through structural, morphological, and thermal analyses. FTIR analysis revealed the dominance of intramolecular beta-sheet structures in wild silks, while the domestic silks and zein favored random coil and alpha-helical structures, respectively. Post-treatments using water annealing further refined the structure and morphology of the films, resulting in stable composites with both inter- and intramolecular beta-sheet structures in wild silks. While in domestic silks, the random coils were converted into intermolecular beta-sheets with enhanced beta-sheet crystallinity. This improvement significantly enhanced the thermal and structural properties of the materials. By deciding on the source, ratio, and treatment of these biopolymers, it is possible to tailor protein blends for a wide range of applications in medicine, tissue engineering, food packaging, drug delivery, and bio-optics.

## 1. Introduction

Materials derived from natural polymers are increasingly being explored across diverse scientific fields, including tissue engineering, materials science, and environmental science. Unlike traditional petroleum- and fossil fuel-based plastics, materials fabricated from naturally derived proteins offer sustainability, broad availability, and cost-effectiveness. Widely studied natural proteins include silk [1], corn zeins [2], soy [3], elastin [4], keratin [5], and collagen [6]. By combining the various properties of these natural biopolymers, materials can be created with specific mechanical, morphological, and thermal properties suitable for a range of applications. In addition, post-processing techniques, such as coagulation baths, can enhance the structural integrity of these materials by regenerating sub-protein structures [7]. This study focuses on characterizing the structural, thermal and micromorphological properties of corn zein and silk, two biopolymers that are widely available from natural sources, economically feasible to manufacture, and hold potential for applications in packaging and the biomedical field. Corn zein is a byproduct of biofuel processing, while silk is produced by various species of spiders and silkworms, with several silkworm species domesticated to facilitate silk production.

Zein, a plant protein derived from corn gluten meal powder, has an alpha-helix structure that imparts distinctive functionalities. Odorless, tasteless, and edible, zein is both biocompatible and biodegradable [8], making it highly suitable for biomedical applications, such as tissue engineering. As a prolamin protein, zein is classified into α, β, γ, and δ subclasses. The α-zein subclass includes two variants, weighing 22 kDa and 24 kDa, respectively, and is composed of repeating α-helix units [9]. The β-zein subclass is smaller, weighing 17 kDa, with a high methionine content, while δ-zein is a minor component at 10 kDa. The γ-zein contains two forms, weighing 27 kDa and 18 kDa. Despite its amphiphilic nature, zein functions as a hydrophobic protein because of its high nonpolar amino acid content [10]. Although the exact structure of zein is still under debate [11,12], it is thought to feature hydrophilic regions at the top, bottom, and core, with a hydrophobic outer surface. This nonpolar amino acid composition enables zein to form stable complexes with drugs [13,14,15,16], underscoring its potential as a drug delivery vehicle. However, zein’s mechanical strength is generally limited [8]: research on crosslinking [17,18] and composite formation with stronger natural polymers [19,20,21] has shown promising strategies to enhance its structural integrity.

Silk is one of the most abundant and strongest natural biomaterials [8,22,23] and consists of 70–80% fibroin and 20–30% sericin by weight, along with small amounts of waxes and inorganic impurities [24]. Silk fibroin (SF), a bioactive protein derived from insects, is widely used in high-quality textiles and offers significant potential for biotechnology, medicine, and pharmaceuticals [8]. SF has also been adapted for use in optics, photonics, electronics, and optoelectronics [8]. At the molecular level, the amino acid structure influences the physicochemical properties of silk. For example, an SF with a high poly-Ala sequence content tends to exhibit greater crystallinity, whereas an SF with predominantly poly-Gly-Ala sequences forms β-sheet structures [8]. The mechanical performance of SF is closely linked to its molecular composition and packing density, with amino acid content and sequence playing critical roles in defining silk’s physical and chemical properties [8].

Despite its remarkable properties, silk alone does not meet all application requirements. To overcome these limitations, SF is often blended with other protein materials to create composites that can be engineered for a wide range of uses [7,25,26,27,28]. These protein-based composites are particularly advantageous in medical research due to their adaptability and ability to be blended to address specific needs [27]. Blending involves mixing different proteins in various ratios to produce an alloy material with properties tailored to specific applications [27]. The resulting material can exhibit unique physical, electrical, chemical, or optical qualities due to strong protein–protein interactions [27], which are influenced by the types, ratios, and processing methods used. Protein molecular structures provide flexibility, enabling attributes such as elasticity, biodegradability, and biocompatibility to be precisely tuned for specific applications, often in the form of films, gels, or fibers [24].

Silkworm silk is obtained from both wild and domesticated sources, leading to biological and physicochemical differences due to environmental factors. This study examines Muga, Tussah, and Eri wild silks, as well as Thai and Mori domestic silks. Thai silk, a naturally occurring biopolymer, is extracted from the cocoons of *Bombyx mori* silkworms in Thailand. Due to environmental influences, Thai silkworm cocoons are yellow and slightly smaller than the white Mori cocoons from China. Traditionally used in textiles, Thai silk proteins have recently been explored for biomedical applications due to their ability to promote cell adhesion and tissue growth [29]. Thai silk’s suitability as a biomaterial is attributed to its mechanical strength, low immunogenicity, thermal stability, and flexibility [29]. Mori silk, derived from *B. mori* silkworms, contains sericin and fibroin. Additionally, these silkworms produce fibers with a heavy and a light chain connected by disulfide bonds and a small P25 protein [30,31]. The choice between wild and domestic silks can be based on their unique structural, chemical, or physical properties, which offer distinct advantages for various applications. Due to their complex intramolecular beta-sheet structures and mechanical stiffness, wild silks, such as Tussah, are renowned for their excellent air permeability, moisture absorption, biocompatibility, and biodegradability. Additionally, they stand out for their superior resistance to heat and corrosion, as well as their strong mechanical properties and ability to support cell adhesion, making them promising materials for tissue regeneration applications [32]. While Muga silk is prized for its natural golden-yellow color, lustrous texture, and the inherent pigments present in its fibers. This silk consists of fibroin surrounded by sericin, a protein with adhesive properties. Muga silk is the strongest of all natural silks, offering exceptional durability, stain resistance, moisture absorption, biodegradability, and biocompatibility. These qualities make it highly valuable in textile applications, decorative items, and biomaterial research, especially in surface modification [33]. Eri silk, another wild silk type, is known for its thermal insulation properties and cost-effectiveness in production. Its affordability and high yield make it a viable option for use in biomaterials and various industrial applications [34]. In contrast, domestic silks, such as Mori and Thai, are commercially available and, predominantly, more flexible, with more coil/helix structures that enable their use in a wide variety of applications, such as biomedical films, textiles, and other engineered materials. For comparison, this study includes Tussah, Muga, and Eri wild silks. Tussah silk, produced by *Antheraea mylitta*, is dark tan; Muga silk, from *Antheraea assamensis*, is light tan; and Eri silk, obtained from *Philosamia ricini* silkworms, is also examined. During spinning, most silk fibers form insoluble crystalline anti-parallel beta-sheets.

Previous studies have primarily focused on either the properties of individual zein or silk materials or domestic silk–zein composites, such as scaffolds with improved antibacterial activity [35] or electrospun nanofibers with good biodegradability [21]. However, their combined potential as blend films has not been fully explored. Our studies on both wild and domestic silks, along with zein, also comprehensively address the structural, morphological, and thermal changes that occur when these two proteins interact. Zein is known for its brittleness and hardness, while silk films are hydrophilic; a combination of zein’s hydrophobicity and silk’s flexibility therefore offers promising prospects for creating biomaterial films. By creating silk–zein hybrid materials in varying ratios, it is possible to fine-tune the structural, morphological, and thermal properties of thin films for targeted applications. Natural biopolymers are crucial in tissue engineering, allowing for the development of materials that are biocompatible and less likely to be rejected by the body. Using biopolymer-based extracellular matrices, it is possible to promote the regeneration of tissues, bones, and even organs.

The three types of wild silk, two types of domestic silk, zein, and hybrids of these silks with zein in seven different ratios were cast into thin protein alloy films and evaluated for their structural, morphological, and thermal properties. Formic acid with calcium chloride (CaCl_2_) was used as a solvent due to its known abilities to enhance mechanical strength [36] and thermal properties [37] in regenerated silk materials. Additionally, the effects following treatment with deionized (DI) water annealing were investigated. Furthermore, our research on zein and various silks lays the foundation for future studies aimed at fine-tuning silk–zein alloy materials for diverse applications. This study also supports sustainability by using renewable biopolymers, as these composites reduce environmental impacts compared to synthetic plastics and promote the use of eco-friendly materials. Zein and zein-based blends exhibit good biodegradability and thermoplastic properties, making them ideal to combine with other polymers. Their amphiphilic nature allows them to blend well with other hydrophilic and hydrophobic polymers [38], thereby improving material performance in the future.

## 2. Results and Discussion

### 2.1. Structural Analysis

**Figure 1:**
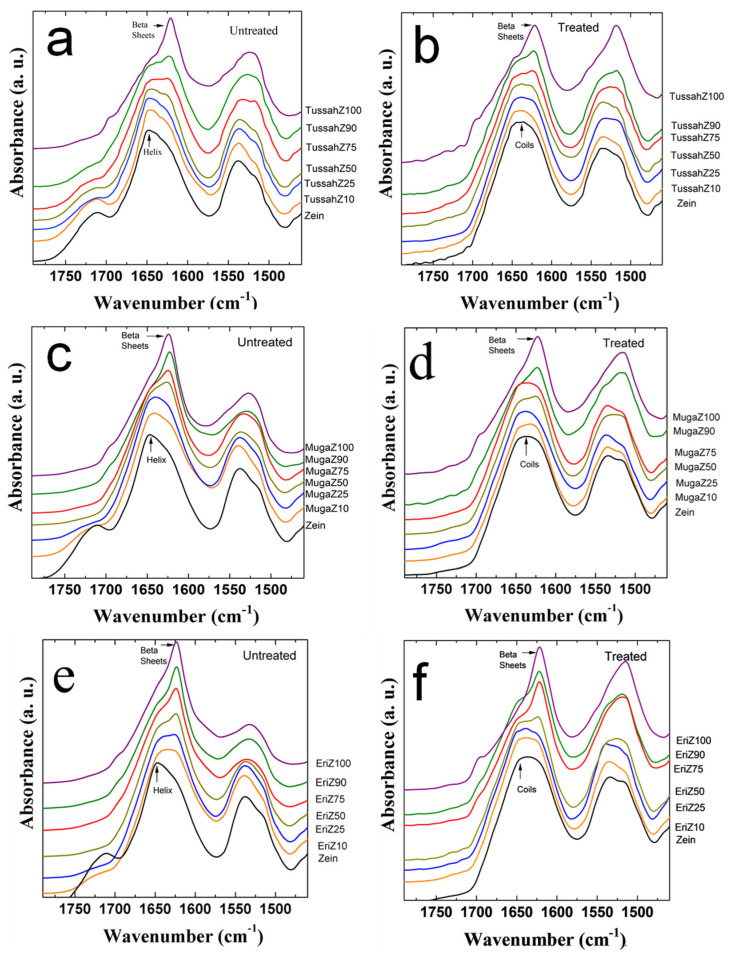
FTIR spectra of films with differing ratios (100%, 90%, 75%, 50%, 25%, 10%, or 0%) of wild-type silks and corn zein before (left) and after (right) water annealing; (**a**,**b**) Tussah–zein (TussahZ), (**c**,**d**) Muga–zein (MugaZ), and (**e**,**f**) Eri–zein (EriZ) silk.

Fourier transform infrared spectroscopy (FTIR) provided insight into the structures of each individual film set. In Figure 1, the Amide I (1600–1800 cm^−1^) and Amide II (1470–1570 cm^−1^) spectra regions of zein–silk blends is shown for varying ratios of silk to zein in the wild silks Tussah–zein (TussahZ) (a, b), Muga–zein (MugaZ) (c, d), and Eri–zein (EriZ) (e, f). The untreated samples are depicted in the left column, while the post-treated (30 min water-annealed) samples are shown in the right column. For untreated samples, pure zein exhibits a dominant alpha-helix structure, as evidenced by the peaks around 1650 cm^−1^ [39]. In contrast, the pure wild silk samples display a mixture of intra- and intermolecular beta-sheet crystalline structures, indicated by peaks around 1622 cm^−1^ [39,40,41]. As the ratio of silk to zein decreases in the hybrid films, the secondary structure increasingly resembles that of corn zein, with a decrease in beta-sheet structures and an increase in alpha-helical structures. This suggests that, by tuning the ratio of silk to zein in each hybrid, the sample structure can be fine-tuned for specific, desired properties.

In the right column of Figure 1, FTIR analysis illustrates how treating the films with water annealing further modifies their structure. After the treatment, zein samples favor a random coil conformation (at 1640 cm^−1^) over an alpha-helical structure, while silks form stronger intermolecular beta-sheets (around 1620 cm^−1^). This is because the treatment washes out Ca^2+^ ions introduced by the solvent, which would otherwise hinder hydrogen bonding and intermolecular interactions, reducing the material’s ability to form more ordered, long-range beta-sheet structures [37]. Zein likely adopts a predominant coil structure, providing a larger surface area for interactions with silk beta-sheets. Since beta-sheets are generally more mechanically and thermally stable than alpha-helices, the composite organizes itself such that the zein networks rely on the beta-sheets from silk. Further evidence for this is seen in the strengthening of the Amide II peak for wild silks, indicating stronger side chain interactions due to molecular interactions between the silk and both itself and the zein [42,43,44]. As the silk-to-zein ratio decreases towards a higher zein content, the films lose their beta-sheet structure and tend to follow a coil structure instead.

**Figure 2:**
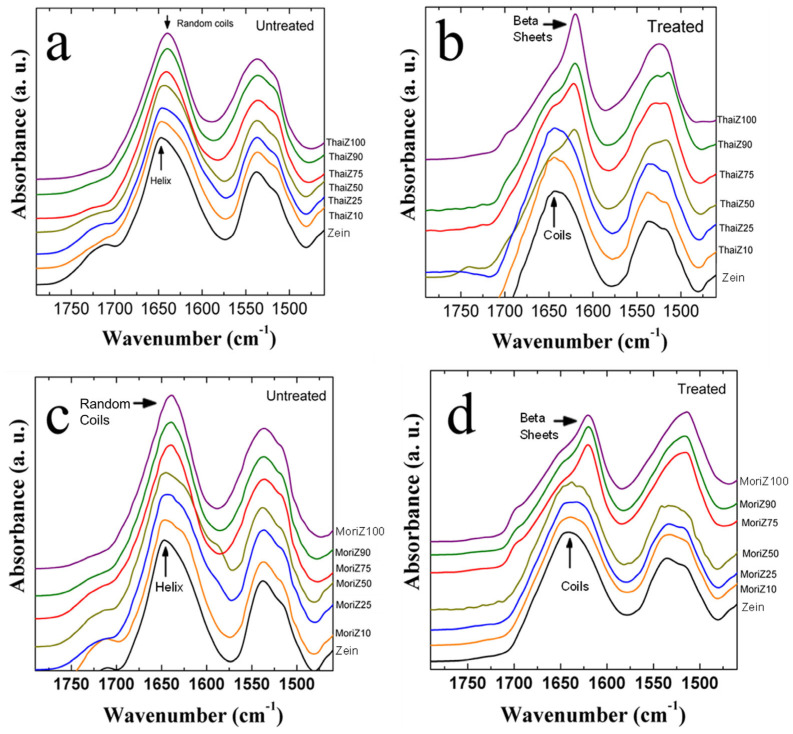
FTIR spectra of domestic silks before (**left**) and after (**right**) treatment by water annealing. The top (**a**,**b**) films are hybrid materials of domestic silks with varying ratios (100%, 90%, 75%, 50%, 25%, 10%, 0%) of Thai silk and zein (ThaiZ), while the bottom (**c**,**d**) films are hybrid materials of Mori silk and zein (MoriZ).

Figure 2 shows the FTIR analysis of silk–zein hybrid films using the domestic silks Thai (a, b) and Mori (c, d). In the domesticated silks, the Amide I region contains peaks in the 1660–1640 cm^−1^ range, indicating the prevalence of alpha-helix (around 1650 cm^−1^), random coils (around 1640–1644 cm^−1^), and intramolecular beta-sheets (around 1637 cm^−1^) [40,45,46,47]. Higher percentages of silk (≥75% silk) were dominated by random coils and intramolecular beta-sheets but gradually shifted to a dominant zein alpha-helical structure (around 1650 cm^−1^) as the composition trended toward a majority of zein. Upon prolonged annealing of silk and silk–zein blends in DI water (right column of Figure 2), calcium ions were eventually removed, disrupting their stabilizing effect on the local intramolecular beta-sheet regions. This promoted the self-assembly of fibroin molecules through hydrogen bonding and hydrophobic interactions, leading to the formation of stronger, stable layer-by-layer intermolecular beta-sheet crystals, which enhanced the films’ water-insolubility, thermal stability, and mechanical integrity. This transition is marked by a decrease in alpha-helices and is evidenced by the silk peaks shifting toward 1620 cm^−1^ [41]. Thus, domestic silks provide an even greater range of crystalline conformations than wild silks, depending on their compositional ratio and post-treatment status. After annealing, beta-sheet-dominated protein materials are well-suited for various biomedical applications.

### 2.2. Thermal Analysis

**Figure 3:**
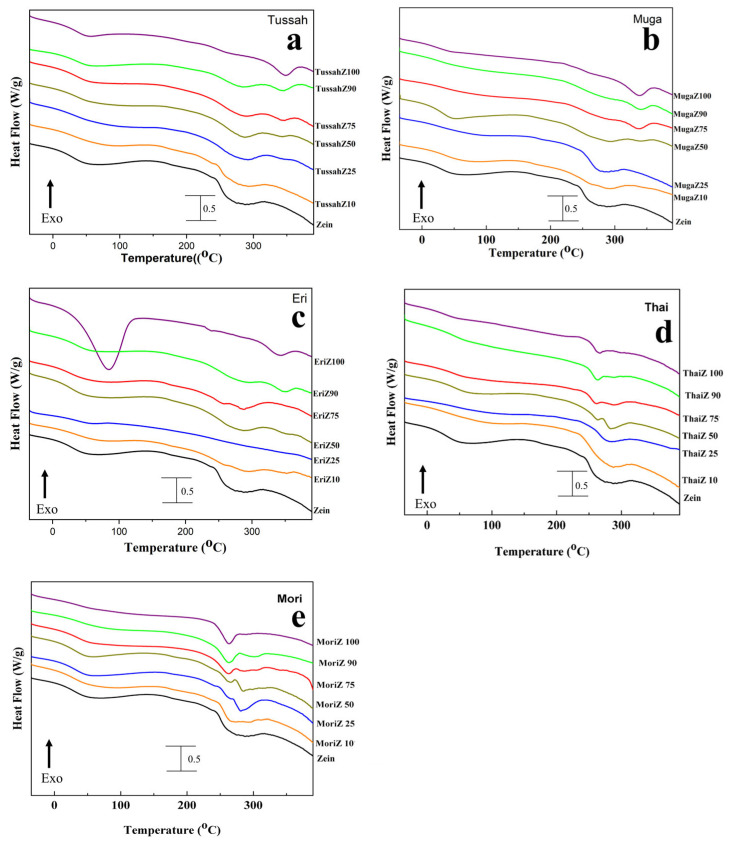
Heat flow (W/g) of silk–zein materials: (**a**) Tussah–zein, (**b**) Muga–zein, (**c**) Eri–zein, (**d**) Thai–zein, and (**e**) Mori–zein films at different silk concentrations (100%, 90%, 75%, 50%, 25%, 10%, 0%).

**Figure 4:**
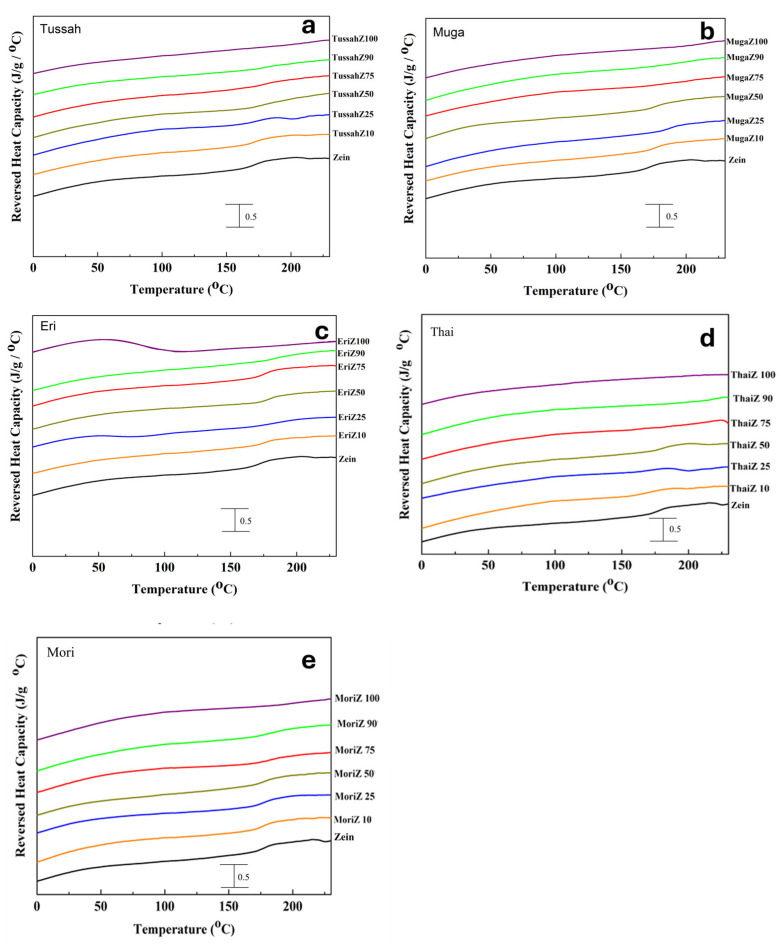
Reversing heat capacity (J/g/°C) of silk–zein materials: (**a**) Tussah–zein, (**b**) Muga–zein, (**c**) Eri–zein, (**d**) Thai–zein, and (**e**) Mori–zein films at different silk concentrations (100%, 90%, 75%, 50%, 25%, 10%, 0%).

Temperature-modulated differential scanning calorimetry (TMDSC) was performed on treated films to better understand the miscibility of the blended protein films and how their composition ratios affected their thermal properties. Figure 3 shows the total heat flow through the protein composite films with different silk concentrations (100%, 90%, 75%, 50%, 25%, 10%, 0%). This test used several degradation temperatures, *T*_d_, obtained from the samples, which are summarized in Table 1, along with the glass transition temperatures, *T*_g_, which were derived from the reversed heat capacity of the samples shown in Figure 4.

In general, all silk proteins have a higher glass transition temperature than zein (171.2 °C), and wild silks typically exhibit a higher glass transition temperature than domestic silks. Therefore, as the zein content increases in the silk–zein composites, their glass transition temperatures gradually decrease. In addition, the higher the silk concentration in the composite, the higher the degradation temperature (except for Thai silk). Pure wild silk films, such as Tussah, Muga, and Eri, exhibit *T*_d_ values near 348.7 °C, 341.3 °C, and 346.2 °C [41], respectively, whereas pure zein films have a *T*_d_ near 273.3 °C [48]. Composite films have one or two *T*_d_ values within this range (Table 1). Interestingly, the domestic silk samples (Mori and Thai) have lower degradation temperatures than their wild counterparts, with *T*_d_ near 265.2 °C and 261.4 °C. This is likely due to the higher thermal stability of wild silk protein films compared to domesticated silk protein films, which is consistent with our previous conclusion [41]. Domesticated silks (Mori and Thai) generally exhibit higher average chain mobility compared to wild silks. This difference in chain mobility may be attributed to structural variations in the proteins. Wild silks potentially have more non-crystalline ordered structures, such as alpha-helices, while domesticated silks (e.g., Thai) tend to have more of the weaker random coil structures. Furthermore, Thai proteins appear to be more susceptible to conformational changes when heated. This structural difference may explain the lower thermal stability of domesticated silks (particularly Thai silks) compared to wild silks, as indicated by DSC studies [42].

Samples with a high silk content tended to show minimal heat capacity increments in the *T*_g_ regions, as the annealing process in deionized water significantly increased beta-sheet crystallinity, resulting in highly crystallized silk proteins with reduced molecular chain mobility during the glass transition [41]. As the silk percentage decreases with added corn zein, the heat capacity increments in the *T*_g_ regions gradually increase, since zein protein chains are not crystallized and have high mobility during *T*_g_. The miscibility of silk and zein in composite films is influenced by their interactions and composition ratio, which govern their thermal properties. The enhanced crystallinity and rigid structures present in wild silks may contribute to improved thermal stability, making such composites suitable for applications like packaging and biomedical materials [42]. Also, the network of beta-sheets in protein structures generally increases rigidity and resistance to mechanical stress, enhancing the biomaterial’s ability to form scaffolds that better mimic the extracellular matrix of hard tissues, supporting cell attachment, proliferation, and differentiation. For example, scaffolds with higher beta-sheet content may offer improved durability and functionality, making them suitable for regenerative medicine, particularly in load-bearing tissues. Conversely, due to their high flexibility, side chain mobility, and processability, domestic silk-based films are ideal for soft tissue engineering or flexible coatings. Therefore, expanding the understanding of molecular interactions between silk beta-sheets and zein alpha-helical structures, and identifying the best composite, could provide deeper insights into how these materials achieve their unique properties for biomedical applications.

### 2.3. Morphological Analysis

**Figure 5:**
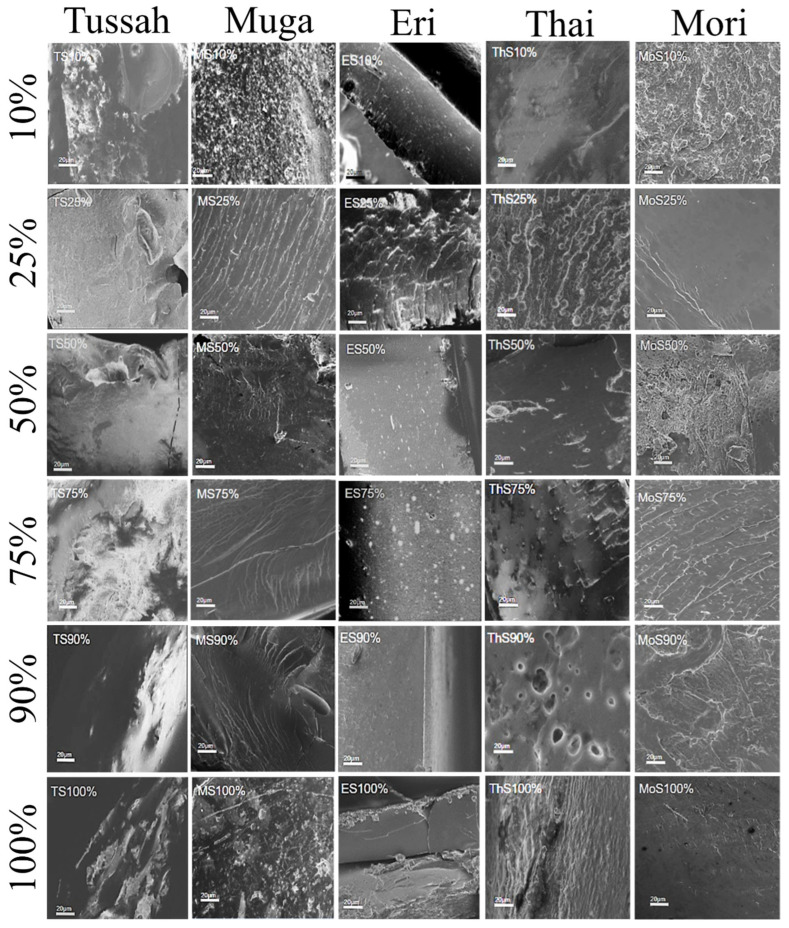
SEM images of silk–zein blended films at low magnification. The blended film compositions, from top to bottom, are 10%, 25%, 50%, 75%, 90%, and 100% silk in the silk–zein films, with Tussah, Muga, Eri, Thai, and Mori silks displayed from left to right. The scale bar in all images represents 20 µm.

**Figure 6:**
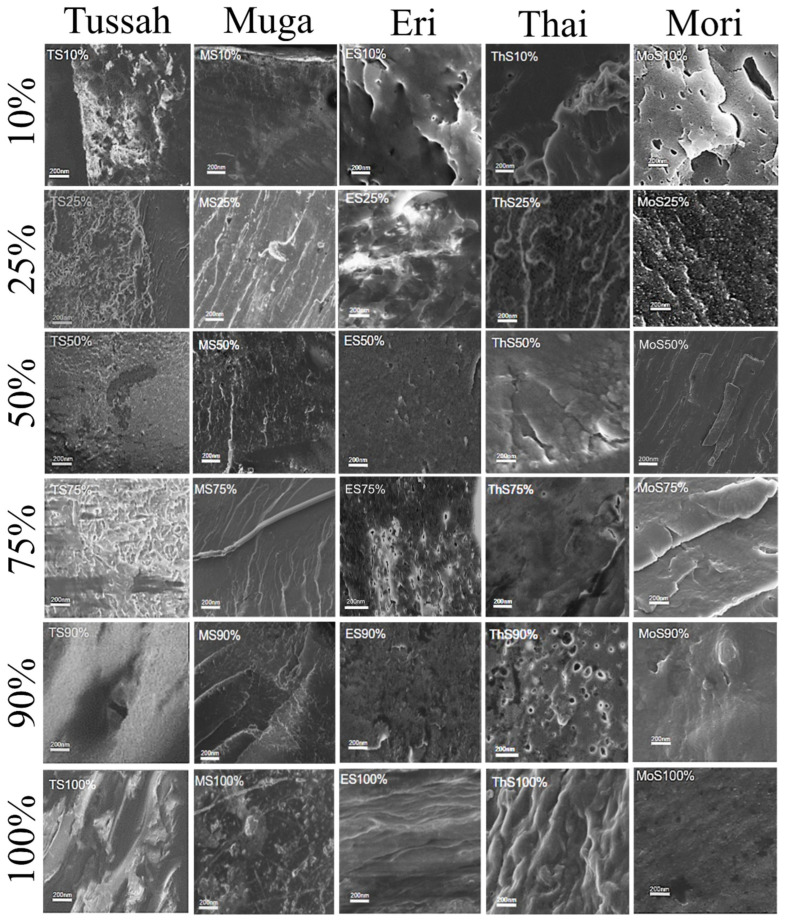
SEM images of silk–zein blended films at high magnification. The blended film compositions, from top to bottom, are 10%, 25%, 50%, 75%, 90%, and 100% silk in the silk–zein films, with Tussah, Muga, Eri, Thai, and Mori silks displayed from left to right. The scale bar in all images represents 200 nm.

**Figure 7:**
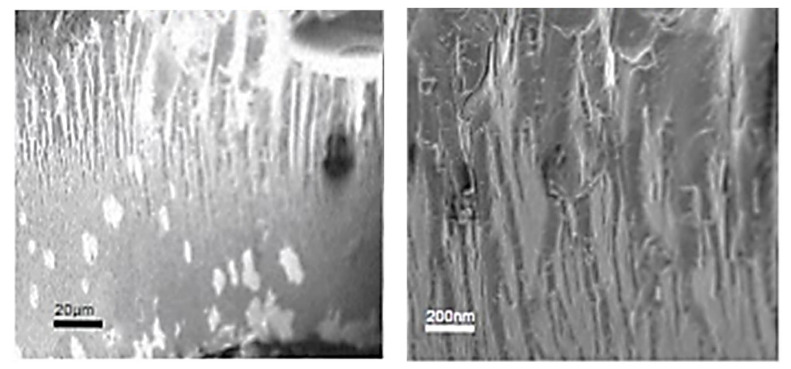
SEM images of a pure zein film with scale bars of 20 µm (**left**) and 200 nm (**right**).

The scanning electron microscopy (SEM) cross-section images in Figure 5 (20 µm scale bar) and Figure 6 (200 nm scale bar) show that the physical blending of silk and corn zein produces a wide variety of micromorphologies in the composite materials. For comparison, Figure 7 shows SEM cross-sectional images of the pure zein film with scale bars of 20 µm and 200 nm. In general, films with high silk levels (≥75%) are less firm, resembling a gel-like soft surface with long-range structures in the cross-section. Films with a higher concentration of corn zein (silk content ≤ 25%) appear firmer and less flexible, displaying more microscale globular structures, which suggests that zein proteins tend to form short-range structures due to the nature of globular proteins. There is a vast diversity of structural features, with notable exceptions, at the 20 μm scale for the cross-sections of silk–corn zein composite films. Muga silk and its composites exhibit particle-like structures, with the composites tending to display smoother features accompanied by linear patterns on the surface. In contrast, Eri silk appears flaky at this scale, while its composites exhibit rougher surfaces. Tussah silk demonstrates increasing roughness at the 20 μm scale, characterized by prominent globular structures, a feature more commonly observed in domesticated silk. Thai silk and its composites show localized patches of roughness at this scale. These differences in structural roughness and component alignment reflect the influence of silk type on the composite’s nanostructure. In addition, these findings demonstrate that, by adjusting the silk-to-zein ratio, the physical and chemical properties of the films can be precisely tailored to meet specific application requirements, offering versatility in the design of biomaterial composites. Overall, it was observed that the cross-sectional images of films tend to be more homogeneous (20 µm scale), with clearer, repeatable microstructures at the 200 nm scale, when the silk source is domestic rather than wild.

### 2.4. Mechanism of Interaction in the Silk–Zein Blends

**Figure 8:**
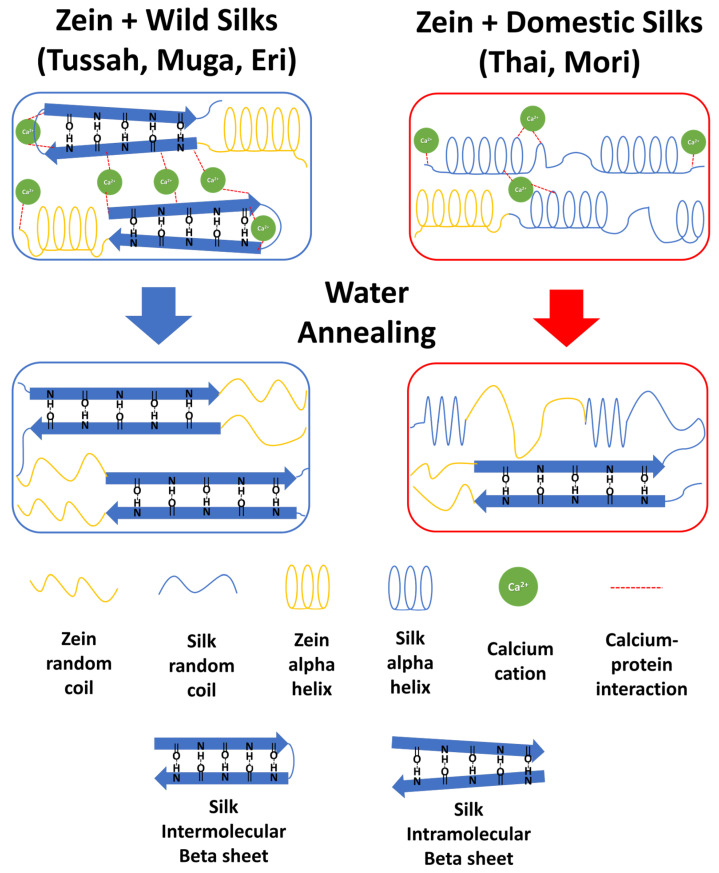
Protein structures of silk–zein blended films before and after the water annealing treatment. The presence of calcium ions inhibits strong beta-sheet crystalline formations.

The structural changes in the silk–zein blended films can be explained by the mechanism illustrated in Figure 8. Based on both FTIR and thermal analyses, there is an observed change in the structural conformation of the proteins in the composite films, depending on their composition ratio and treatment status. In Mori and Thai (domestic) silks, the protein conformation shifts from random coils and alpha-helices to intermolecular beta-sheet crystalline structures after treatment with DI water to remove Ca^2^⁺ ions, as indicated by a shift in the Amide I peak from 1660–1640 cm^−1^ to 1620 cm^−1^ in the FTIR analysis. Wild silks also form stronger long-range intermolecular beta-sheet crystals from local intramolecular beta-sheets after the treatment. The structure of these films can also be altered by incorporating more globular zein proteins into the silk matrix, which shifts their original helix structure to random coils following water annealing. This change is also seen in the DSC analysis, where the degradation peak and glass transition temperature trend downward with an increase in zein due to the weaker thermal integrity of zein random coils compared to silk beta-sheet crystals. Zein’s random coil structure, however, provides a larger surface area for intermolecular interactions with silk. This is observed as a widening of the Amide I peak, indicative of the protein backbone, and a sharpening of the Amide II peak, indicative of side chain movement. In untreated films, the presence of calcium ions disrupts the material’s ability to interact through hydrogen bonding, hindering intermolecular interactions. After treatment, however, silk can form strong intermolecular beta-sheets with itself and interact with the loose random coil structure of zein.

## 3. Materials and Methods

### 3.1. Preparation of Materials

**Figure 9:**
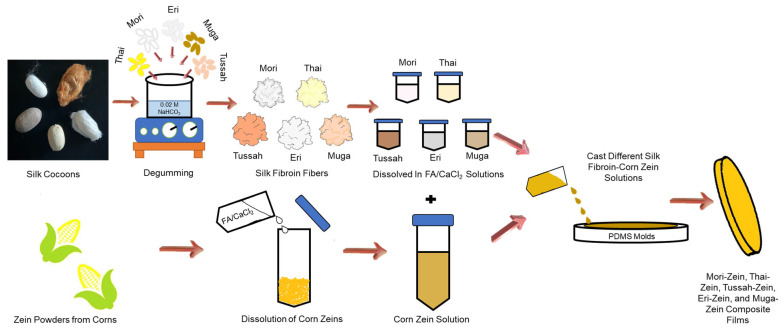
Fabrication procedures of corn zein–silk composite films.

The three types of wild-type silks, *Philosamia ricini* (Eri), *Antheraea assamensis* (Muga), and *Antheraea mylitta* (Tussah) were obtained from India as cocoons. *Bombyx mori* silk cocoons were obtained from two separate domestic sources: from China (Mori) and Thailand (Thai). Prior to processing the silks into films, sericin was removed from the cocoons in order to extract the silk fibroin, following previous protocols [42,49]. Briefly, this was accomplished by boiling cocoons in 0.02 M NaHCO_3_ solution for 2 h, followed by three deionized water washes (Figure 9). Silk fibroin was then dried for 24 h in a 60 °C oven. Purified corn zein protein powders (Zein) were obtained from POET, LLC (Sioux Falls, SD, USA). Formic acid of 98% purity (ACS grade) and calcium chloride (CaCl_2_) were purchased from EMD Millipore Corporation (Burlington, MA, USA). Calcium chloride was used to create a 4 wt% solution with formic acid in order to dissolve the silks and zein.

Silk–zein films were created by first dissolving the individual silks into tubes of about 17.5 mL using a solution of formic acid with CaCl_2_. The zein was then dissolved in a separate 17.5 mL vial with the same formic acid-CaCl_2_ solution (Figure 9). Once separately dissolved, the two solutions were mixed to acquire blends of silk and zein (0%, 10%, 25%, 50%, 75%, 90%, and 100% weight ratios). Finally, the solutions were poured onto polydimethylsiloxane (PDMS) molds and left to dry for approximately 24 h in a fume hood (Figure 9). Excess formic acid was removed by drying the films in a vacuum oven for 24 h at room temperature (25 °C) under 10 millibars of pressure while ensuring consistent humidity levels. All films were dried under the same environmental conditions to minimize variations in the outcome. The final untreated films had a thickness of approximately 20 µm. The thickness was controlled by evenly casting the film onto the PDMS mold. For treated samples, films were then annealed in deionized water for 30 min and dried overnight (12 h) in a fume hood at room temperature.

### 3.2. Fourier Transform Infrared Spectrometry (FTIR)

Data was collected using a Bruker Tensor 27 Fourier Transform Infrared Spectrometer (Billerica, MA, USA) equipped with a deuterated triglycine sulfate detector and a multiple reflection horizontal MIRacle ATR attachment (using a Ge crystal from Pike Tech, Madison, WI, USA). IR spectra were captured from 4000 cm^−1^ to 400 cm^−1^ at a resolution of 4 cm^−1^. Prior to any run, a background scan was performed, followed by 64 sample scans with duplicate runs (*n* > 4) taken on each side of the film. This was followed by preprocessing, which included smoothing and normalization to improve data quality. The average spectrum from each sample was analyzed to highlight the effects of different ratios and water annealing treatments on film morphology. The ATR crystal was cleaned with compressed air between each run to remove any residues.

### 3.3. Scanning Electron Microscopy (SEM)

Cross-sectional images of the blended films were taken using a Phenom Pure scanning electron microscope (Eindhoven, The Netherlands) with an EHT of 10 kV. The samples were frozen with liquid nitrogen and then broken into approximately 1 cm^2^ squares using tweezers while submerged in liquid nitrogen. The samples were attached to black double-sided carbon tape and placed onto a sample holder. Prior to imaging, all samples were sputter coated with gold for 15 s using a Denton Vacuum Desk II sputtering machine (Moorestown, NJ USA) in order to improve their conductivity. Images were collected at an accelerating voltage of 5~10 kV at magnifications of 1000× and 30,000×. The images were taken at different locations across the cross-sectional surface of the film to confirm homogeneity.

### 3.4. Temperature-Modulated Differential Scanning Calorimetry (TM-DSC)

Samples of each film (about 6 mg) were put into aluminum pans before they were heated in a TA Instruments Q100 DSC (New Castle, DE, USA), which was purged with a dry nitrogen gas flow (50 mL/min) and equipped with a refrigerated cooling system. Samples were heated from −40 °C to 400 °C at a rate of 2 °C/min, with a modulation period of 60 s and temperature amplitude of 0.318 °C. Samples with a diameter of approximately 2.5 mm were cut from the original films and tested three times for each condition. Prior to testing, the instrument was calibrated using an indium standard for heat flow. The instrument was calibrated to heat capacity using aluminum and sapphire standards.

## 4. Conclusions

This study compares five types of silk (Mori, Thai, Muga, Tussah, and Eri) fabricated into films combined with plant-based zein proteins. When silk comprised the majority of the film, the domestic silk blend films of Mori–zein and Thai–zein showed a structural shift from a random coil-dominated structure to strong intramolecular beta-sheets after water annealing; however, when zein was predominant, an alpha-helical structure was maintained. Water treatment caused zein to adopt a random coil structure, enhancing its interaction with silk’s beta-sheets and promoting intramolecular bonding. In contrast, wild silk blends (Tussah, Muga, and Eri) initially contained weak intramolecular beta-sheets that strengthened following the removal of calcium ions from the solvent during treatment. A similar effect was observed in wild silk blends as the zein content increased. The thermal analysis using DSC showed that adding corn zein protein lowered the glass transition and degradation temperature peaks of the composite (except in Thai silk). Morphological analysis revealed that higher zein content corresponded to an increase in nanoscale, short-range globular structures linked to zein’s helix/random coil structure, contributing to films with a reduced mechanical integrity characteristic of zein proteins. The wild silk composites retained their beta-sheet structures, especially when the silk content exceeded the zein content. By adjusting the silk-to-zein ratio, selecting the silk source, and applying a water treatment, a broad range of biocomposite material properties can be tailored for specific applications. The limitations of silk–zein composites, such as moisture sensitivity and challenges in processing due to the differing solubility characteristics of wild silks, as well as their mechanical and biological properties, will be addressed in the future. In comparison to other biopolymer systems, like collagen or chitosan-based composites, silk–zein composites may lack some properties like excellent biocompatibility [50], yet they remain a viable option due to their cost-effectiveness and ease of processing for certain applications.

## Figures and Tables

**Table 1:** Thermal characteristics of silk–zein protein films at various ratios (100%, 90%, 75%, 50%, 25%, 10%, 0% silk) representing wild silks (Tussah, Muga, Eri) and domestic silks (Thai, Mori). All values have an error bar within ±5%.

Sample	Silk Composition /wt%	*T*_g_/°C	Degradation 1 *T*_d_/°C	Degradation 2 *T*_d_/°C	Reference
TussahZ 100	100	233.9	348.7	N/A	[41]
TussahZ 90	90	179.9	294.1	344.5	
TussahZ 75	75	178.6	290.8	346.4	
TussahZ 50	50	177.6	289.2	346.4	
TussahZ 25	25	177.4	287.5	346.4	
TussahZ 10	10	177.2	280.69	344.8	
MugaZ 100	100	214.7	341.3	N/A	[41]
MugaZ 90	90	195.3	275.9	341.9	
MugaZ 75	75	189.5	288.3	339.3	
MugaZ 50	50	182.9	291.1	339.8	
MugaZ 25	25	180.8	277.8	N/A	
MugaZ 10	10	178.9	266.3	291.8	
EriZ 100	100	238.1	346.2	N/A	[41]
EriZ 90	90	185.3	294.1	349.3	
EriZ 75	75	180.8	290.1	N/A	
EriZ 50	50	178.9	287.5	350.6	
EriZ 25	25	177.1	288.7	351.5	
EriZ 10	10	174.1	287.3	353.4	
ThaiZ 100	100	217.3	265.2	N/A	[41]
ThaiZ 90	90	202.3	268.1	N/A	
ThaiZ 75	75	194.2	268.3	288.2	
ThaiZ 50	50	183.5	270.1	284.8	
ThaiZ 25	25	176.8	283.5	N/A	
ThaiZ 10	10	176.8	282.3	N/A	
MoriZ 100	100	183.9	261.4	N/A	[41]
MoriZ 90	90	183.1	263.3	298.3	
MoriZ 75	75	182.1	265.4	289.8	
MoriZ 50	50	181.3	268.3	285.1	
MoriZ 25	25	180.1	262.1	283.0	
MoriZ 10	10	179.2	249.1	278.8	
Zein	0	171.2	273.3	277.7	[48]

## Data Availability

The original contributions presented in this study are included in the article. Further inquiries can be directed to the corresponding author(s).
